# Reduced graphene oxide-encaged submicron-silicon anode interfacially stabilized by Al_2_O_3_ nanoparticles for efficient lithium-ion batteries†

**DOI:** 10.1039/d4ra00751d

**Published:** 2024-04-09

**Authors:** Xiangyu Tan, Zhongqiang Zhao, Zhimin Na, Ran Zhuo, Fangrong Zhou, Dibo Wang, Longchang Zhu, Yi Li, Shaocong Hou, Xin Cai

**Affiliations:** a Power Science Research Institute of Yunnan Power Grid Co., Ltd Kunming 650214 China; b School of Electrical Engineering and Automation, Wuhan University Wuhan 430072 China sc.hou@whu.edu.cn; c College of Materials and Energy, South China Agricultural University Guangzhou 510642 China caixin2015@scau.edu.cn; d Qujing Power Supply Bureau of Yunnan Power Grid Co., Ltd Qujing 655099 China; e Electric Power Research Institute, China Southern Power Grid Guangzhou 510623 China

## Abstract

Silicon–carbon composites have been recognized as some of the most promising anode candidates for advancing new-generation lithium-ion batteries (LIBs). The development of high-efficiency silicon/graphene anodes through a simple and cost-effective preparation route is significant. Herein, by using micron silicon as raw material, we designed a mesoporous composite of silicon/alumina/reduced graphene oxide (Si/Al_2_O_3_/RGO) *via* a two-step ball milling combined annealing process. Commercial Al_2_O_3_ nanoparticles are introduced as an interlayer due to the toughening effect, while RGO nanosheets serve as a conductive and elastic coating to protect active submicron silicon particles during lithium alloying/dealloying reactions. Owing to the rational porous structure and dual protection strategy, the core/shell structured Si/Al_2_O_3_/RGO composite is efficient for Li^+^ storage and demonstrates improved electrical conductivity, accelerated charge transfer and electrolyte diffusion, and especially high structural stability upon charge/discharge cycling. As a consequence, Si/Al_2_O_3_/RGO yields a high discharge capacity of 852 mA h g^−1^ under a current density of 500 mA g^−1^ even after 200 cycles, exhibiting a high capacity retention of ∼85%. Besides, Si/Al_2_O_3_/RGO achieves excellent cycling reversibility and superb high-rate capability with a stable specific capacity of 405 mA h g^−1^ at 3000 mA g^−1^. Results demonstrate that the Al_2_O_3_ interlayer is synergistic with the indispensable RGO nanosheet shells, affording more buffer space for silicon cores to alleviate the mechanical expansion and thus stabilizing active silicon species during charge/discharge cycles. This work provides an alternative low-cost approach to achieving high-capacity silicon/carbon composites for high-performance LIBs.

## Abstract 2

The cycling stability of ball milled silicon/graphene composite can be improved by an interlayer of Al_2_O_3_ nanoparticles for lithium storage.

## Graphical abstract

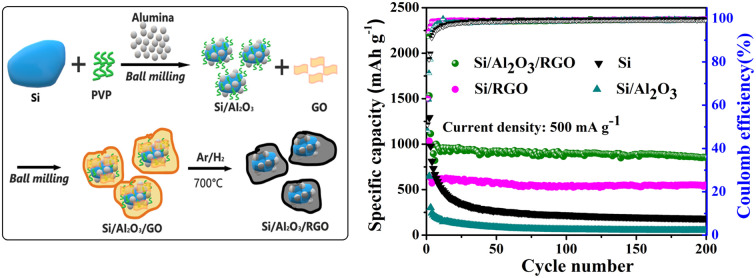

## 1 Introduction

Since their first commercialization in the early 1990s, rechargeable lithium-ion batteries (RLIBs) with high specific energy density and low pollution have been widely used in communication equipment, mobile electronic devices, plug-in hybrid/electric vehicles and many other fields.^1^ After more than 30 years of rapid development, current RLIBs based on common graphite anodes and layered cobalt-based oxide (*e.g.* LiCoO_2_) cathodes are almost close to their theoretical energy density limit.^2^ The exploration of novel high-efficiency anode and cathode materials is essential to further improve the energy output and multiscale operating safety of LIBs.^3,4^ For example, the theoretical capacity of the graphite anode is only 372 mA h g^−1^; therefore, developing more efficient anode materials is a viable strategy to promote the current LIB performance further.^5^ In particular, silicon-based anode materials stand out as some of the most prospective anode candidates in regard to their high theoretical capacity of 4200 mA h g^−1^, moderate redox potential window (<0.5 V *vs.* Li^+^/Li) for alloying/dealloying reactions, sufficient earth reserve and environmental friendliness.^6,7^ Nevertheless, silicon anodes often suffer from poor electronic conductivity and drastic volumetric expansion (>300%)/contraction during the lithiation/delithiation process, which result in an exceptionally unstable solid electrolyte interface (SEI) on electrode surfaces and the stress-induced pulverization of active silicon particles on the current collector, thus seriously harming battery capacity, rate capability and long cycling life.^8^

Apart from using more suitable electrolytes or robust binders, previous studies have shown that nanostructurization and porous engineering are fundamental strategies for constructing efficient silicon anodes.^9–11^ Primarily, silicon materials with rational nanoarchitecture and porosity can mitigate mechanical strains and buffer the volumetric deformation of active silicon species during lithium intercalation and deintercalation reactions, leading to the greatly reduced critical fracture size and prolonged cycling stability of silicon.^12^ Also, both the ionic diffusion and mass transfer kinetic can be accelerated throughout the nano-silicon or three-dimensional (3D) porous silicon electrode for improved rate performance and cyclic tolerance.^13^ To further boost the reliable cycling of the silicon anodes, carbon coating/modification of silicon particles is an extremely conducive and widely adopted way.^14,15^ Such hybridization with carbonaceous components undoubtedly endows the silicon/carbon composites with enhanced conductivity, mechanical strength and more void space for inhibited electrode expansion upon charge/discharge due to the conductive and mechanically strong carbon layers.^16^ Up to now, numerous carbon materials, including mesoporous carbon, graphite, carbon nanotubes (CNTs), graphenes, pyrolytic amorphous carbon, and carbon nanofibers, have been used to modify silicon and developed as high-capacity composite anodes for RLIBs, exhibiting improved cycling capacities and increased battery life.^16–21^

Among those carbonaceous materials, two-dimensional (2D) graphene nanosheets are attractive by virtue of good conductivity and ionic diffusivity, high specific area, chemical/thermal stability and superior mechanical properties.^22^ Hence, silicon–graphene composites with elaborate architecture and reasonable composition are promising to boost high-performance silicon anodes.^23–25^ For instance, Zhou *et al.* dispersed silicon nanoparticles (NPs) between graphene layers through an electrostatic attraction-guided self-assembly route and obtained stable anode with increased cycling performance.^26^ Taking advantage of the CNTs current collector and the atomically thin graphene capping layer prepared from DC sputtering, graphene-draped thick silicon films obtained an average capacity of 806 mA h g^−1^ over 1000 cycles, benefiting from the monolayer graphene sheath to stabilize the SEI films formed at the silicon/electrolyte interfaces.^27^ Serving as a conductive agent, hollow graphene particles were found to effectively alleviate the volumetric expansion of silicon, showing a thickness expansion of 20.4% in the fully-lithiation state over 200 cycles.^28^ Recently, Lu and coworkers developed a graphene-supported double-layer carbon-wrapped Si anode, which was synthesized from metal–organic frameworks, sucrose and graphene oxide precursors using solvothermal reaction and high-temperature pyrolysis. The resulting anode achieved a high capacity of 1182 mA h g^−1^ with 89.5% retention over 240 cycles at 0.2 A g^−1^.^29^ Despite the great progress of existing advanced silicon–graphene anode composites, using more cost-effective resources such as commercial micron silicon or silicon oxide particles rather than expensive silicon NPs will be even appealing toward practical application.^30^ Besides, many reported high-efficiency silicon/graphene composites are still limited by complicated and energy-consuming preparations such as vapor deposition, physical sputtering, electrospinning/spraying, template technique, or wet-chemical process.^27,31–34^ It is urgently necessary to explore more convenient, facile and scalable synthetic strategies to construct uniform and high-efficiency silicon–graphene anodes toward practical RLIBs.^35^

Previous studies have revealed that a suitable Al_2_O_3_ thin layer can help inhibit the formation and excessive growth of SEI films, leading to improved electrochemical stability of LIBs, even though the introduction of Al_2_O_3_ into efficient silicon anodes through low-cost and simple fabrication remains difficult.^36,37^ To deal with the above concerns, here, we proposed a novel anode composite of silicon/alumina/reduced graphene oxide (Si/Al_2_O_3_/RGO), which was prepared with a micron silicon resource by a two-step ball milling assisted process. For the first time, commercial γ-type Al_2_O_3_ was introduced to fabricate efficient silicon anode materials. The constructed Si/Al_2_O_3_/RGO was mesoporous and composed of submicron silicon particles interfacially coated with Al_2_O_3_ NPs as well as wrapped by thin RGO layers. By profiting from the high specific area of the rational porous structure, conductive RGO matrix, and especially the dual protection of the Al_2_O_3_ interlayer and the robust RGO shells to withstand the structure deformation of silicon upon lithiation/delithiation process, Si/Al_2_O_3_/RGO was endowed with high activity, excellent conductivity, favorable electrolyte diffusion and interfacial charge transfer, and structural integrity that are suitable for lithium storage. When used as the anode, the Si/Al_2_O_3_/RGO composite delivered a high initial discharge capacity of 2202 mA h g^−1^ under the current of 100 mA g^−1^ with an initial coulombic efficiency of 63%. After 200 charge/discharge cycles at 500 mA g^−1^, Si/Al_2_O_3_/RGO can still maintain a high reversible capacity of 852 mA h g^−1^. Also, Si/Al_2_O_3_/RGO demonstrated superb rate capabilities with a stable discharge capacity of 405 mA h g^−1^ at a high current density of 3000 mA g^−1^, significantly superior to the Si/RGO counterpart.

## 2 Experimental section

### 2.1 Synthesis of the anode composites

Preparation of graphene oxide (GO): GO was synthesized from commercial microsized graphite flakes, according to a reported study.^38^ Preparation of Si/Al_2_O_3_/RGO composites: the composites were basically obtained by two-step ball milling of micron silicon powder (40–200 mesh, Aladdin), alumina (γ type, 10 nm, Aladdin) and GO. As for the first-step ball milling, 500 mg of micron silicon, alumina (50 mg, 100 mg and 200 mg, respectively) and 100 mg of polyvinylpyrrolidone (PVP, average molecular weight of 360 000) were added into the polytetrafluoroethylene jar of a planetary ball mill, followed by the addition of 10 mL of absolute ethanol. The total mass of the agate beads was 90 g; among them, the mass ratio of the 5 mm bead to the 10 mm bead was maintained at 1 : 1. The planetary ball mill operated at a speed of 500 rpm for 12 h to form the Si–Al_2_O_3_ suspension. For the second ball milling process, 500 mg of graphene oxide powder was added into the Si–Al_2_O_3_ suspension and continued for another 12 h ball milling at the same speed and other conditions. The obtained slurry was dried in a blast drying box under 80 °C, which was further transferred into the tube furnace and calcined at 700 °C for 2 h at the heating rate of 3 °C min^−1^ with the protection of an Ar/H_2_ (95/5) atmosphere. Finally, Si/Al_2_O_3_/RGO (50), Si/Al_2_O_3_/RGO (100), and Si/Al_2_O_3_/RGO (200) composites were respectively obtained when the sample was cooled to room temperature. Unless otherwise noted, Si/Al_2_O_3_/RGO (100) was denoted as Si/Al_2_O_3_/RGO in this work. Meanwhile, the comparative composites of Si/RGO (without Al_2_O_3_ addition) and Si/Al_2_O_3_ (without GO inclusion) were also obtained through the same procedures.

### 2.2 Material characterizations

The morphology and the fine structure of these involved materials (Si/Al_2_O_3_/RGO, Si/RGO, Si/Al_2_O_3_, and Si) were investigated by field-emission scanning electron microscopy (FE-SEM, Merlin, Zeiss) and high-resolution transmission electron microscopy (HR-TEM, JEOL-2010). The phase compositions of these samples were obtained by X-ray powder diffractometer (XRD, Rigaku) implementing the Cu Kα radiation at 40 kV at a scanning speed of 10° min^−1^. Raman spectrograms were recorded using a LabRam HR spectrometer (Aramis, France), applying the laser excitation line of 532 nm across the range of 300–2000 cm^−1^. Thermogravimetric analysis (TGA) was performed on a Netzsch analyzer under air conditions. The specific surface area and the pore distribution of composites were determined by analyzing the nitrogen adsorption–desorption behavior at 77 K by a Gemini-2360 apparatus. The X-ray photoelectron spectroscopy (XPS) characterization of the Si/Al_2_O_3_/RGO composite was conducted for the study of surface chemical composition.

### 2.3 Device assembly and electrochemical measurements

To evaluate the electrochemical performance of these samples, CR2025-type coin cells were assembled in the recirculating argon glove box where the contents of water and oxygen are below 0.1 ppm. The coil cell used lithium metal as the counter electrode. The working electrodes were fabricated by coating homogeneously blended slurries, which contain the active material (80 wt%, *e.g.* Si/Al_2_O_3_/RGO, Si/RGO, Si/Al_2_O_3_, and Si), the conducting agent (acetylene carbon, 10 wt%), and the polymeric binder (sodium alginate, wt.10%) dissolving in deionized water, onto the copper foil substrate. After coating, the electrodes were dried in a vacuum oven at 80 °C for 12 h. The mass loading of the active electrode was 1.0–1.1 mg cm^−2^. 1.0 M lithium hexafluorophosphate (LiPF_6_) was dispersed in a mixed solvent, including dimethyl carbonate (DMC) and ethylene carbonate (EC), with a volumetric ratio of 1 : 1 to form the electrolyte. A piece of polymeric microporous monolith (Celgard 2400) was selected as the separator. For studying the performances of those electrode materials in terms of cycling capacities, rate performance and galvanostatic charge/discharge profiles, a Neware battery system was utilized across the voltage range of 0.01–1.2 V (*vs.* Li/Li^+^) under a constant temperature of 25 °C and different current densities. All mentioned specific capacities were calculated according to the total weight of the active material, such as the Si/Al_2_O_3_/RGO composite. The cyclic voltammetry (CV) behavior of all the samples was studied by a CHI660C electrochemistry workstation over the voltage window of 0.01–1.2 V, adopting the sweep speed of 0.2 mV s^−1^. The electrochemical impedance spectroscopy (EIS) curves were gained by setting the frequency cutoff from 100 kHz to 0.1 Hz by exerting the amplitude disturbance of 5 mV on the coin cells.

## 3 Results and discussion

**Fig. 1:**
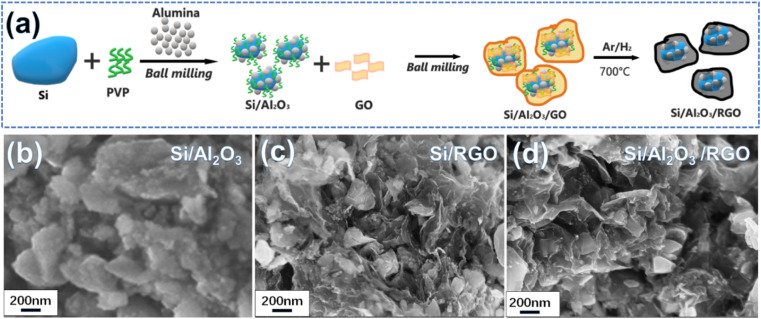
(a) Schematic of the preparation process of the Si/Al_2_O_3_/RGO composite from micron silicon powder. SEM image of the as-synthesized Si/Al_2_O_3_ (b), Si/RGO (c) and Si/Al_2_O_3_/RGO (d).

**Fig. 2:**
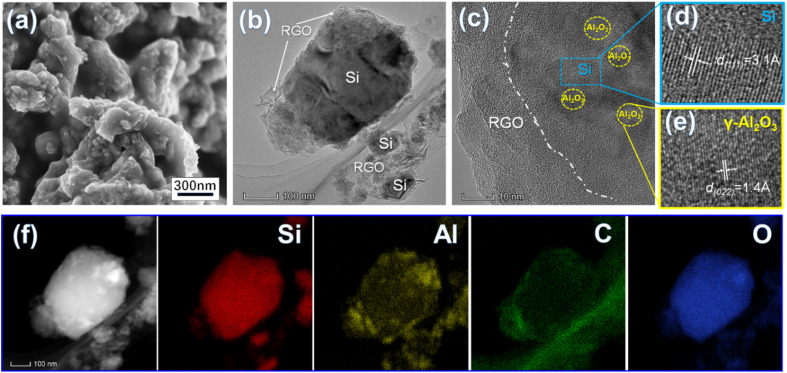
SEM image (a) and HR-TEM images (b–e) of the Si/Al_2_O_3_/RGO composite and its elemental mapping images (f).

Fig. 1a schematically depicts the two-step ball milling-assisted preparation of the Si/Al_2_O_3_/RGO composites. During the first ball milling process, micron silicon (m-Si), PVP and alumina were evenly mixed *via* mechanical milling. In this regard, m-Si particles were drastically down-sized to irregular and submicron nanoparticles with smooth surfaces (Fig. S1a and b†). Meanwhile, tiny Al_2_O_3_ nanoparticles could be well spread over the surfaces of the Si particles with the auxiliary PVP adhesives under rigorous blending (Fig. 1b). Notably, PVP molecules not only serve as the polymeric adhesive to bind the components but also act as surfactants to inhibit the agglomeration of active silicon particles during high-energy ball milling.^39^ Subsequently, GO nanoflakes were added for another ball milling to form homogeneous nanocomposites. After the thermal treatment of the ball-milled precursor at 700 °C, the final Si/Al_2_O_3_/RGO composites were obtained. SEM images in Fig. 1d and 2a demonstrate the porous morphology of the Si/Al_2_O_3_/RGO composite, where submicron silicon particles are distinctly covered with thin and semitransparent RGO layers. In comparison, the Si/RGO sample (Fig. 1c, S1c and d†) exhibits a similar morphology to that of Si/Al_2_O_3_/RGO, suggesting that the interlayer Al_2_O_3_ nanoparticles have no obvious impact on the routine morphology of the composites and RGO is effective in wrapping silicon through such a ball milling process.

High-resolution transmission electron microscopy (HR-TEM) characterization further identifies the fine structure of Si/Al_2_O_3_/RGO. As shown in Fig. 2b, structurally intact silicon particles with an average size of 200–300 nm are near-elliptic, and the surfaces are all-around covered with flexible RGO nanosheets. According to Fig. 2c–e, a general core/shell structure of Si/Al_2_O_3_/RGO is observed, with the inner silicon core and the multilayer RGO sheathes. The lattice spacing of 0.14 nm corresponds to the (022) crystal plane of γ-type Al_2_O_3_ phase, while the spacing of 0.31 nm is index to the (111) plane of crystalline Si,^40^ which verifies the existence of tiny Al_2_O_3_ nanocrystals distributing between the interlayers of silicon particles. In addition, energy dispersive spectroscopy (EDS) mapping analyses clarified the elemental composition of the Si/Al_2_O_3_/RGO composite (Fig. S2†). As presented by the distribution of Si, Al, C and O elements in Fig. 2f, silicon particles are dually wrapped by Al_2_O_3_ nanoparticles and the RGO layers, further indicating the Al_2_O_3_ embedded and rationally core/shell structured Si/Al_2_O_3_/RGO composite as mentioned above.

**Fig. 3:**
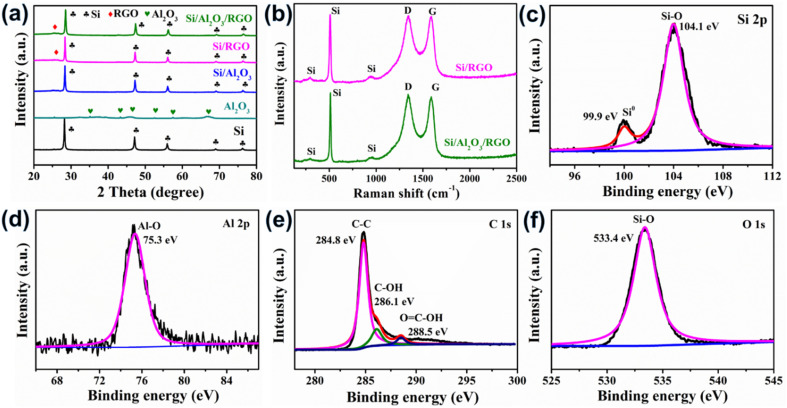
(a) XRD patterns of ball milled Si, Al_2_O_3_, Si/RGO, Si/Al_2_O_3_ and Si/Al_2_O_3_/RGO. (b) Raman spectra of the composites. XPS spectra of Si/Al_2_O_3_/RGO. (c) Si 2p spectrum. (d) Al 2p spectrum. (e) C 1s spectrum. (f) O 1s spectrum.

The phase structures of as-synthesized samples were analyzed by X-ray diffraction (XRD), as illustrated in Fig. 3a. The sharp peaks centered at 2*θ* = 28.3°, 47.2°, 56.1°, 69.0° and 76.2° are assigned to the featured planes of (111), (220), (311), (400), and (331) from the silicon crystallites (PDF# 27-1402), which can be found in Si, Si/Al_2_O_3_, Si/RGO, and Si/Al_2_O_3_/RGO as expected.^41^ Compared with pure Si, the peaks associated with the silicon phase become apparently weakened in the composites, which is attributed to the inclusion of Al_2_O_3_ and RGO coating. The appearance of a weak blunt peak at 2*θ* = 26.5° in the Si/RGO and Si/Al_2_O_3_/RGO composites can be ascribed to the (002) plane of crystallized graphites originating from the RGO component in the composite.^42^ In contrast to pure Al_2_O_3_, it is difficult to clearly distinguish the set of characteristic Al_2_O_3_ peaks in Si/Al_2_O_3_ and Si/Al_2_O_3_/RGO due to the relatively weak diffraction peaks and considerably low proportion of Al_2_O_3_ compared to the strong silicon peaks. Besides, there are no obvious impurity peaks in Si, Si/Al_2_O_3_, Si/RGO and Si/Al_2_O_3_/RGO, which means negligible byproducts generated during the synthetic process.

Fig. 3b displays the Raman spectra of the as-received materials. The three characteristic peaks centered at 295 cm^−1^, 509 cm^−1^ and 939 cm^−1^ stem from the crystalized silicon phase in both Si/Al_2_O_3_ and Si/Al_2_O_3_/RGO composites.^43^ The peaks located at 1348 cm^−1^ and 1587 cm^−1^ correspond to the D band and the G band of the carbon component, which are correlated with the disordered carbon defects and the graphitic crystallites of carbonaceous materials, respectively. As for the Si/Al_2_O_3_/RGO composite, the intensity ratio of the D band and the G band (*I*_D_/*I*_G_) is determined to be 1.03, and the value is 0.998 for the Si/RGO composite. The higher *I*_D_/*I*_G_ of the Si/Al_2_O_3_/RGO composite than that of Si/RGO is mainly attributed to the increased edge defects of graphene nanosheets, which may result from more disordered grain boundaries due to the incorporation of tiny alumina nanoparticles during the ball milling synthesis. Generally, the increased amorphous carbon defects could provide isotropic channels for the diffusion of lithium ions and also benefit the volumetric accommodation of the active silicon species during repeated cycling.^44^ Brunauer–Emmett–Teller (BET) measurement is carried out to characterize the porosity of the materials. According to the nitrogen adsorption/desorption isotherms and pore-size distribution curves in Fig. S3a and b,† the BET specific area and total pore volume of Si/Al_2_O_3_/RGO are 106.11 m^2^ g^−1^ and 0.194 cm^3^ g^−1^, higher than those of Si/RGO (43.93 m^2^ g^−1^ and 0.169 cm^3^ g^−1^), respectively. Besides, Si/Al_2_O_3_/RGO owns an average pore width of 12.2 nm, which is smaller compared to Si/RGO (20.8 nm). The larger specific surface area and mesoporous structure of Si/Al_2_O_3_/RGO with plentiful pores can not only ensure the available contact area between the silicon-based electrode and the electrolyte for ion diffusion but also favor the alleviation of mechanical stress caused by the silicon expansion/contraction during lithiation/delithiation process.^45^ Moreover, thermal gravimetric (TG) analysis is conducted in the air to determine the proportion of carbon/silicon components in the composites (Fig. S4a and b†). Results demonstrate that both Si/RGO and Si/Al_2_O_3_/RGO contain high silicon content of over 60 wt% along with carbon species of *ca.* 33.6 wt%.

X-Ray photoelectron spectroscopy (XPS) characterization of the Si/Al_2_O_3_/RGO composite was carried out to investigate its surface chemical composition. As shown in Fig. 3c, there are two distinct peaks appearing at 99.9 eV and 104.1 eV, which exactly correspond to the Si^0^ (monatomic silicon) and the high-valent Si^4+^ species (from Si–O bonding), respectively. Obviously, the prominent peak of Si–O bonding should be caused by the produced SiO_*x*_ layers shielding around the surfaces of silicon due to the unavoidable oxidation of pristine silicon during the high energy ball milling under atmospheric conditions.^46,47^ It is reasonable that a minor SiO_*x*_ sheath may help to improve the contact between silicon and the polar RGO nanosheets, thereby benefiting the establishment of uniform Si-GO composites. Through the Al 2p spectrum displayed in Fig. 3d, the peak at 75.3 eV corresponds to the Al–O bonding from the Al_2_O_3_ constituent in Si/Al_2_O_3_/RGO. Fig. 3e shows the C 1s spectrum, where three deconvoluted peaks are attributed to the sp^2^-hybridized C–C bonds and the oxygen-containing functional groups on the surface of RGO nanosheets. The sharp and dominant peak of C–C bonding also indicates the high degree of reduction of the GO precursor to form the conductive RGO component in Si/Al_2_O_3_/RGO, favoring improved electrical conductivity and good charge transport.^48^ Meanwhile, the peak occurring at 533.4 eV in the O 1s spectrum is assigned to the Si–O species (Fig. 3f). All the above results confirm the construction of a mesoporous Si/Al_2_O_3_/RGO composite that is basically composed of RGO layers and Al_2_O_3_-protected submicron silicon particles.

**Fig. 4:**
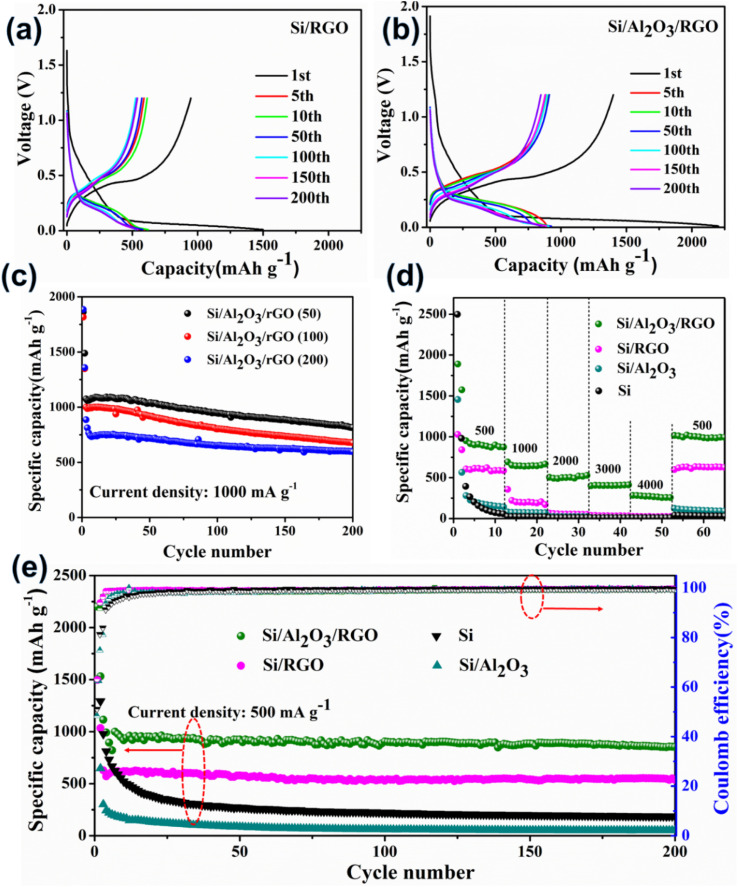
Galvanostatic voltage-capacity curves of Si/RGO (a) and Si/Al_2_O_3_/RGO (b) electrode materials at different cycles. (c) Cycling performance of different Si/Al_2_O_3_/RGO electrodes (different feed/content of Al_2_O_3_ involved in the synthesis) under 1000 mA g^−1^. The first two cycles are measured at 100 mA g^−1^. (d) Discharge capacities of the electrode materials at different current densities (500 mA g^−1^, 1000 mA g^−1^, 2000 mA g^−1^, 3000 mA g^−1^ and 4000 mA g^−1^). (e) Cycling performance of different electrodes and corresponding coulombic efficiencies under 500 mA g^−1^, among which the initial two cycles are measured at 100 mA g^−1^.

Primarily, we assembled coil-type half cells to evaluate the electrochemical performance of these silicon-based anode materials. Fig. 4a, b, S5a and b† present the galvanostatic voltage-capacity curves of pure Si, Si/Al_2_O_3_, Si/RGO and Si/Al_2_O_3_/RGO at different cycles between 0.01–1.2 V at a current density of 500 mA g^−1^. All the silicon-based electrode materials exhibit almost similar galvanostatic voltage-capacity behaviors due to the dominant active silicon species. As such, the voltage plateaus observed across 0.3–0.5 V in the charging curves imply the lithium deintercalation from the Li_*x*_Si alloy phase, while the long voltage plateaus at 0.15–0.05 V in the discharging curves are commonly formed by the intercalation reaction of Li^+^ into the crystallized Si species.^49^ Compared to the pure silicon and Si/Al_2_O_3_ electrodes, Si/Al_2_O_3_/RGO and Si/RGO demonstrate excellent electrochemical activity and obviously more stable cyclic performance with highly overlapped charging/discharging platforms during repeated cycles. The initial discharge-specific capacity of the Si/Al_2_O_3_/RGO composite is 2202 mA h g^−1^ with an initial coulombic efficiency of 63%. The irreversible capacity loss can be mainly ascribed to the intensive electrolyte decomposition correlated with the production of solid electrolyte interface (SEI) between the electrode and the electrolyte during the first lithiation process.^50,51^ In contrast, Si/RGO, Si/Al_2_O_3_, and pure silicon electrodes deliver an initial discharge capacity of 1498 mA h g^−1^, 1494 mA h g^−1^, and 2577 mA h g^−1^, corresponding to an initial coulombic efficiency of 63%, 48% and 51%, respectively. Significantly, the dramatically improved initial discharge capacities and considerable coulombic efficiency of Si/Al_2_O_3_/RGO are synergistically contributed by the RGO shells and Al_2_O_3_ interlayer, which could provide more accessible pathways for electron transport and lithium diffusion/reaction, as well as effective structural protection of the vulnerable silicon electrodes. Also, the impact of Al_2_O_3_ loading on the electrochemical performance of the Si/Al_2_O_3_/RGO composite was studied. As displayed in Fig. 4c, the three Si/Al_2_O_3_/RGO samples with different Al_2_O_3_ feeds demonstrate impressive cycling capacities even under a high current density of 1000 mA g^−1^. Notably, excessive Al_2_O_3_ loading could harm the cycling capacities of Si/Al_2_O_3_/RGO since those electrochemical inert and redundant Al_2_O_3_ nanoparticles may obstruct/interfere with the available active sites for lithium electrochemistry. Although Si/Al_2_O_3_/RGO (50) seems to obtain the highest capacities, the insufficient reproducibility of the low alumina-loaded composite remains a problem. Therefore, Si/Al_2_O_3_/RGO (100) is ultimately preferred for optimized Li^+^ storage.

The rate performances of different anode materials are illustrated in Fig. 4d. The discharge current was set by gradually increasing from 500 mA g^−1^ to 4000 mA g^−1^ every ten cycles and finally recovered to 500 mA g^−1^. Accordingly, the Si/Al_2_O_3_/RGO electrode achieved an average reversible capacity of 908 mA h g^−1^ (500 mA g^−1^), 640 mA h g^−1^ (1000 mA g^−1^), 501 mA h g^−1^ (2000 mA g^−1^), 405 mA h g^−1^ (3000 mA g^−1^), and 272 mA h g^−1^ (4000 mA g^−1^), significantly superior to those of the Si/RGO, Si/Al_2_O_3_, and Si electrodes. When the current returns to 500 mA g^−1^, the Si/Al_2_O_3_/RGO electrode restored a considerable discharge capacity of 990 mA h g^−1^, manifesting outstanding rate capabilities and high reversibility. For comparison, the Si/RGO electrode showed a discharge capacity of only 605 mA h g^−1^ at 500 mA g^−1^, which quickly drops to 200 mA h g^−1^ as the current rises to 1000 mA g^−1^, even though the performance outperforms the inferior Si/Al_2_O_3_ and the silicon electrodes as expected. Fig. 4e shows the cycling performances and corresponding coulombic efficiencies of different electrodes upon continuous charge/discharge cycles at 500 mA g^−1^. After the first few cycles, the specific capacities of Si/Al_2_O_3_/RGO can be well kept due to dynamically stabilized SEI films, along with the gradual electrode activation upon continuous cycles. After 200 cycles, Si/Al_2_O_3_/RGO achieved high coulombic efficiencies of above 99.3% and can still maintain a high specific capacity of 852 mA h g^−1^, giving rise to an average decay rate of 0.026% per cycle and high capacity retention of about 85%, suggesting the remarkably enhanced cycling stability and electrochemical reversibility of Si/Al_2_O_3_/RGO compared to Si/RGO and the other counterparts. It is concluded that the dual protection from the Al_2_O_3_ interlayer and the RGO coating could effectively buffer the volumetric change of the active silicon during the Li^+^ insertion/extraction process, leading to improved cycling capacities, high-rate performance and excellent durability.

**Fig. 5:**
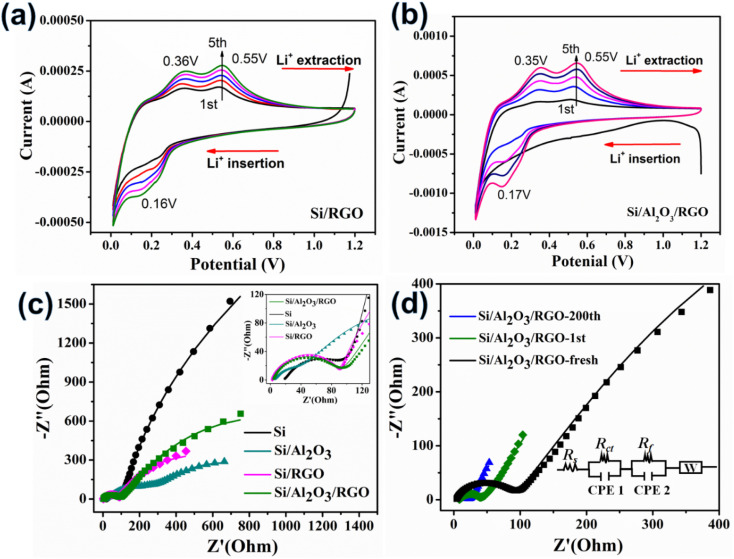
Cyclic voltammograms of Si/RGO (a) and Si/Al_2_O_3_/RGO (b). (c) Fitted EIS plots of different electrodes. Inset: enlarged view of the high-mid frequency region. (d) Fitted EIS plots of Si/Al_2_O_3_/RGO at different cycles (fresh, 1st cycle and 200th cycle). Inset: the equivalent circuit for EIS fitting.

Electrochemical analyses of half cells were further performed to investigate the lithiation/delithiation reaction process of the electrodes. Fig. 5a and b present the cyclic voltammetry (CV) curves of Si/RGO and Si/Al_2_O_3_/RGO over the voltage window of 0.01–1.2 V using a scan rate of 0.2 mV s^−1^, respectively. Typically, the characteristic cathodic peaks at 0.16–0.17 V are generated by the lithium insertion reaction of Si to form Li_*x*_Si alloys, while the two-anodic characteristic peaks at *ca.* 0.35 V and 0.55 V belong to the lithium extraction from the Li_*x*_Si phase back to Si species,^52^ in accordance with the aforementioned charge/discharge voltage plateaus. Note that the gentle protrusion appearing at 0.5–0.75 V in the first cathodic scan is related to the intensive construction of the SEI layers surrounding the active electrode in the initial period, which generally causes some irreversible structural change of the active material.^53^ The symbolic redox peaks of Si/Al_2_O_3_/RGO and Si/RGO electrodes become stronger as the cycle number increases. Such a phenomenon not only reflects the sluggish activation of the porous and active silicon electrodes but also indicates complete and stable SEI films on the silicon surfaces with rational protection of RGO coating.^54^ Among all the silicon-based electrodes, the highest peak currents and continuously strengthened redox peaks of Si/Al_2_O_3_/RGO further reveal its superb Li^+^ storage activity and reversibility, shedding light on the synergistic contribution of the Al_2_O_3_ interlayer and the RGO thin sheath to improve the silicon–lithium reactivity and the electrochemical tolerance of the composite electrode upon redox cycles. In contrast, the CV curves of Si/Al_2_O_3_ and pure Si electrodes show similar lithiation/delithiation peaks but visibly weaker currents and attenuated retention ability of the peaks as compared to Si/Al_2_O_3_/RGO and Si/RGO (Fig. S6a and b†), mainly due to the dynamically unstable SEI films on the silicon particles being overexposed to the organic electrolytes.^55,56^

Furthermore, the interfacial reaction kinetic of the electrodes was probed by electrochemical impedance spectroscopy (EIS) characterization. The Nyquist plots of different electrodes were modeled and elucidated in Fig. 5c. In common, *R*_s_ expresses the intrinsic ohmic resistance, and *R*_ct_ corresponds to the interfacial charge transfer resistance of the active electrode.^50^ For all the RGO-containing electrodes, the *R*_s_ values (2.9 Ω for Si/RGO and 3.9 Ω for Si/Al_2_O_3_/RGO) are significantly smaller than that of pure Si electrode (18.9 Ω), resulting from the highly conductive RGO component. The excellent electronic conductivity of Si/Al_2_O_3_/RGO and Si/RGO is essential to ensure fast Li^+^ storage so as to realize the desirable cycling performance and rate capability discussed above. More importantly, Si/Al_2_O_3_/RGO demonstrates a much lowered *R*_ct_ of 86.1 Ω compared to Si (7823 Ω) and Si/Al_2_O_3_ (816.6 Ω). It is reasonable that those insulating Al_2_O_3_ nanoparticles can favor the interfacial charge transfer dynamic, although at the expense of electrical conductivity to some extent. Fig. 5d displays the EIS plots of the Si/Al_2_O_3_/RGO electrode under different cycles. As compared to the fresh electrode, *R*_ct_ of the cycled electrode (18.7 Ω after the first cycle and 4.83 Ω after 200 cycles) dramatically decreased, probably originating from the progressive electrolyte activation and well-shaped SEI films, benefiting interfacial charge transfer and ionic diffusion throughout the electrode.^57,58^ Besides, *R*_s_ clearly increased for the 1st-cycled electrode (7.1 Ω) and then reduced for the 200th-cycled electrode (4.6 Ω), which further suggests the initial construction of the SEI layer during the first cycle and gradual stabilization of the dynamic SEI film on the silicon surfaces of Si/Al_2_O_3_/RGO during the repeated lithiation/delithiation process. These results clarify that our Si/Al_2_O_3_/RGO electrode possesses high electric conductivity, accelerated charge transfer and ion diffusion as well as prominent structural integrity, thus achieving excellent electrochemical performance upon charge/discharge cycling.

**Fig. 6:**
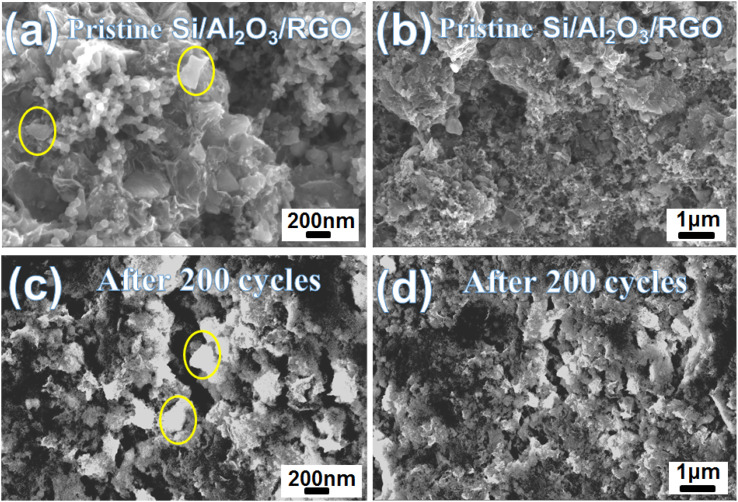
SEM images of the Si/Al_2_O_3_/RGO electrode before (a and b) and after (c and d) 200 charge/discharge cycles at 500 mA g^−1^.

To graphically recognize the structure retention, the morphology variation of the Si/Al_2_O_3_/RGO electrode after intensive charge/discharge cycles at 500 mA g^−1^ was studied. As shown in Fig. 6a and b, before cycling, a large number of wrinkled translucent RGO nanosheets and small carbon black particles can be distinguished in the as-prepared Si/Al_2_O_3_/RGO electrode. After 200 charge/discharge cycles, rough lithium layers are uniformly coated on the surfaces of the electrode. It is encouraging that the pristine RGO-wrapped structure and porous morphology of Si/Al_2_O_3_/RGO remained intact without apparent particle pulverization or fall-off from the current collector.^59^ Therefore, the dual protection strategy of Al_2_O_3_ interlayer and RGO coating could well suppress the volumetric expansion of the submicron silicon particles and alleviate the excessive growth of the SEI films during the prolonged Li^+^ alloying/dealloying process. In addition, conductive and elastic RGO nanosheets can evidently promote the poor conductivity of silicon and ensure tight connections between the active particles for more efficient Li^+^ storage.

## 4 Conclusion

In summary, we have constructed mesoporous Si/Al_2_O_3_/RGO composites through a simple synthetic route combining two-step mechanical ball milling and thermal post-treatment. Core/shell structured Si/Al_2_O_3_/RGO was composed of submicron silicon particles that were dually protected by Al_2_O_3_ NPs interlayer and the intact RGO nanosheet layers, which could favor more available active specific area for electrolyte penetration, improved electrical conductivity, reduced charge transfer resistance, and especially structural robustness of the silicon-based electrode. Results also revealed the synergistic contribution of the RGO shell and Al_2_O_3_ NPs to improve the interfacial toughening of the composite, thus accommodating the volumetric expansion of silicon upon the continuous lithium insertion and extraction process. When used as anode material for Li^+^ storage, the Si/Al_2_O_3_/RGO electrode obtained high initial specific capacity, a stable reversible capacity of 852 mA h g^−1^ at a current density of 500 mA g^−1^ after 200 charge/discharge cycles, and excellent rate capabilities under different current densities of up to 5000 mA g^−1^, demonstrating superior cycling durability that outperformed the Si/RGO counterpart. In light of the low-cost resource, simple and scalable synthesis, and impressive electrochemical performance, the well-constructed Si/Al_2_O_3_/RGO composites in this study may offer a viable strategy to achieve cost-efficient and practical anode materials for new-generation high-energy LIBs.

## Conflicts of interest

The authors declare no conflict of interest.

## Supplementary Material

RA-014-D4RA00751D-s001
