# Supplementary material for: Reduced graphene oxide-encaged submicron-silicon anode interfacially stabilized by Al2O3 nanoparticles for efficient lithium-ion batteries
Source: RSC Adv. 2024 Apr 9;14(16):11323–33. doi: 10.1039/d4ra00751d (PMC11002566; doi:10.1039/d4ra00751d)
Supplement: RA-014-D4RA00751D-s001 [file RA-014-D4RA00751D-s001.pdf]

Electronic Supplementary Material (ESI) for RSC Advances.
This journal is © The Royal Society of Chemistry 2024

Supporting information

Reduced Graphene Oxides Encaged Submicron-silicon Anode Interfacially Stabilized by Al_2O_3 Nanoparticles for Efficient Lithium-ion Batteries

Xiangyu Tan [1], Zhongqiang Zhao [3], Zhimin Na [4], Ran Zhuo [5], Fangrong Zhou [1], Dibo Wang [5], Longchang Zhu[1], Yi Li [2], Shaocong Hou [2,*] Xin Cai[3,*]

[1] *Power Science Research Institute of Yunnan Power Grid Co., Ltd, Kunming 650214, China*
[2] *School of Electrical Engineering and Automation, Wuhan University, Wuhan 430072, China*
[3] *College of Materials and Energy, South China Agricultural University, Guangzhou 510642, China*
[4] *Qujing Power Supply Bureau of Yunnan Power Grid Co., Ltd, Qujing 655099, China*
[5] *Electric Power Research Institute, China Southern Power Grid, Guangzhou 510623, China*

**Corresponding author E-mail address:*
sc.hou@whu.edu.cn (Shaocong Hou)
caixin2015@scau.edu.cn (Xin Cai)

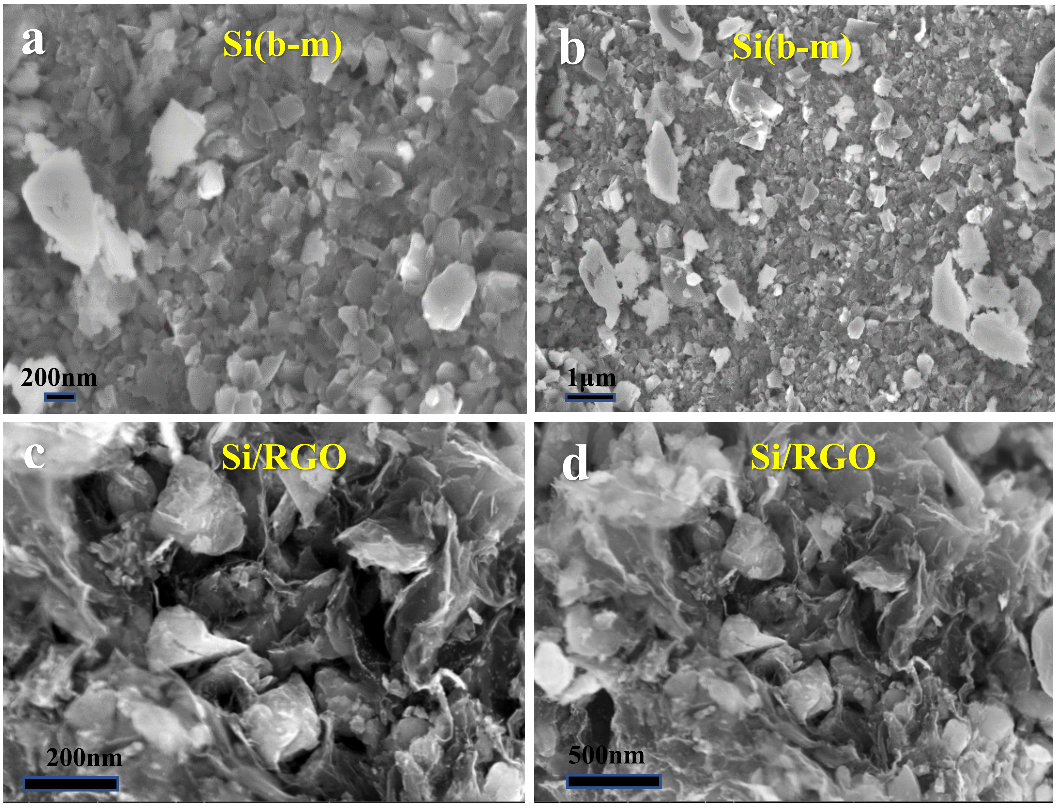

Figure S1 SEM images of ball milled Si (a, b) and Si/RGO (c, d).

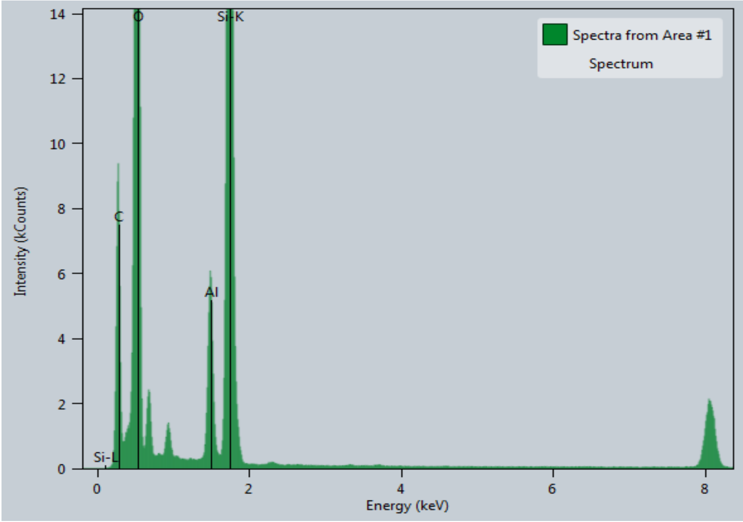

Figure S2 Energy dispersion spectrum of $Si/Al_2O_3/RGO$.

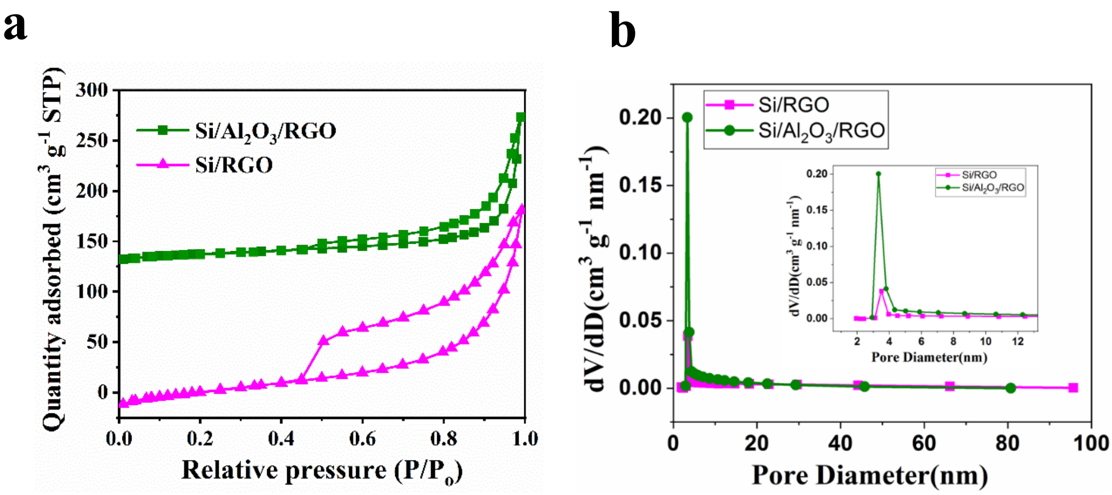

Figure S3 N_2 adsorption/desorption isotherms at 77 K (a) and the relevant BJH pore-size distribution of Si/RGO and $Si/Al_2O_3/RGO$ composites.

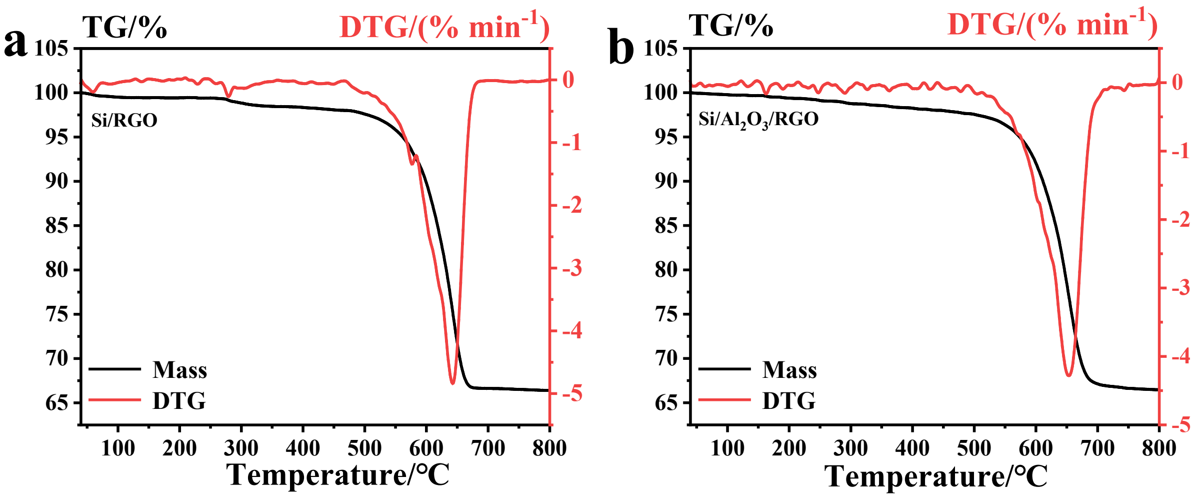

Figure S4 TGA curves of Si/RGO (a) and $Si/Al_2O_3/RGO$ (b).

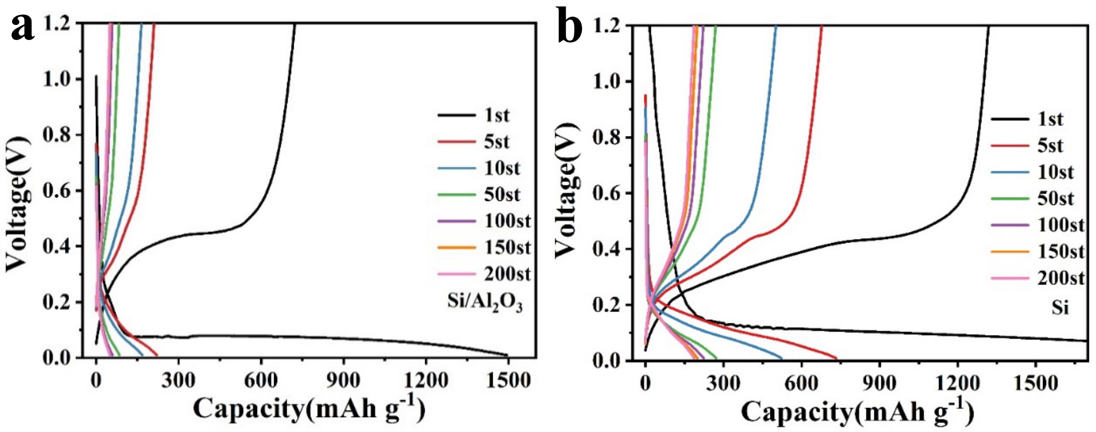

Figure S5 Galvanostatic voltage-capacity curves of Si/Al_2O_3 (a) and pure silicon (b) electrode materials at different cycle (1st, 5th, 10th, 50th, 100th, 150th and 200th cycles).

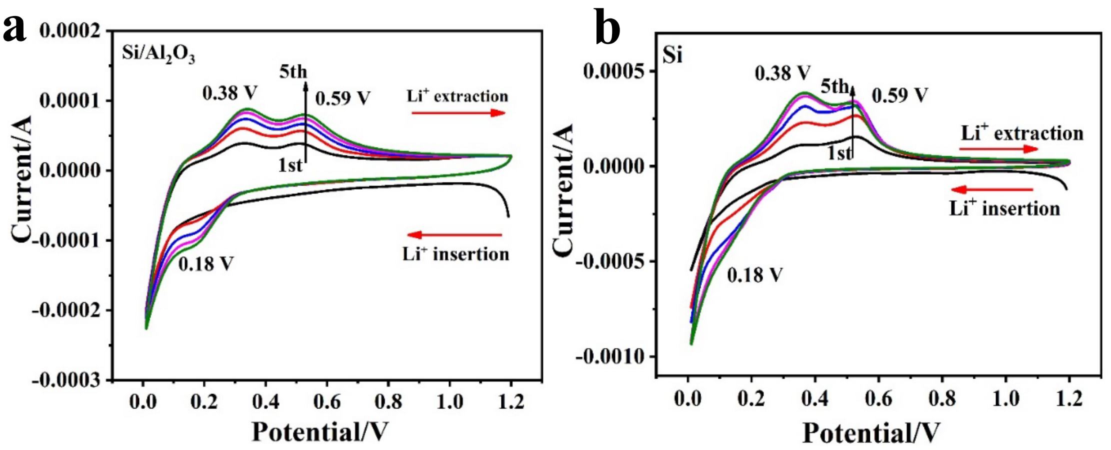

Figure S6 Cyclic voltammograms of the Si/Al_2O_3 electrode (a) and pure Si electrode (b).
